# Intrapleural Tissue Plasminogen Activator and Dornase Alfa Administration for a Multiloculated Recurrent Malignant Pleural Effusion: A Case Report

**DOI:** 10.7759/cureus.24373

**Published:** 2022-04-22

**Authors:** Kia Nikoomanesh, Alexander T Phan, Veerpal Sond, Mufadda Hasan

**Affiliations:** 1 Pulmonary and Critical Care, Arrowhead Regional Medical Center, Colton, USA; 2 Internal Medicine, Arrowhead Regional Medical Center, Colton, USA

**Keywords:** pulmonary adenocarcinoma, intrapleural therapy, dornase alfa, tissue plasminogen activator (tpa), malignant pleural effusion

## Abstract

Malignant pleural effusions (MPEs) can often be very difficult to manage despite conservative interventions including thoracentesis and indwelling pleural catheter placement. These effusions can be septated and loculated, leading to complexities in drainage and symptomatic relief for patients. As such, physicians have experimented with the use of tissue plasminogen activator (t-PA) and dornase alfa (DNase) in attempts to drain complex malignant pleural effusions. Although the use of t-PA and DNase has been well studied in the context of empyema, the literature is limited in regards to the use of these medications in MPEs. Here, we present the case of a patient with a history of metastatic lung adenocarcinoma complicated by recurrent MPEs. Bedside ultrasonography revealed a septated fluid pocket in the pleural space of the right hemithorax. An indwelling pleural catheter (IPC) was placed with minimal symptomatic relief. The decision was made to administer t-PA and DNase through the IPC, resulting in the resolution of symptoms and radiographic findings. This case highlights the potential benefit of using t-PA and DNase to help drain complex malignant pleural effusions.

## Introduction

Malignant pleural effusions (MPEs) are defined by an accumulation of exudate and cancerous cells in the pleural space [1]. MPEs are a common complication of advanced cancer, with an incidence of greater than 150,000 cases per year, and are associated with increased healthcare costs, given the rising rates of cancer worldwide [1,2]. Patients with MPEs can have widely varying clinical presentations: some patients present asymptomatic, while others experience significant acute respiratory distress [2]. Management of MPEs has been a topic of heavy debate over the past few decades. Interventions for MPEs are also quite variable, depending on patient symptomatology, rate of reaccumulation, and patient prognosis [1,2]. These interventions include therapeutic thoracentesis, tube thoracostomy, mechanical or chemical pleurodesis, and placement of an indwelling pleural catheter (IPC) [1,2]. While there are many options available, there has been a recent shift in the management of MPEs toward minimally invasive procedures geared toward symptomatic relief and improved patient-reported outcomes [1]. 

In 2018, the American Thoracic Society (ATS) recommended initiating management of a suspected symptomatic MPE with therapeutic thoracentesis. Thoracentesis was recommended to help clarify whether a patient's symptoms were specifically related to MPE and if the lung would appropriately expand following pleural drainage. This same 2018 guideline also recommended the placement of an IPC or chemical pleurodesis for more definitive therapy in the management of dyspnea in patients with MPE and confirmed expandable lungs. Furthermore, they also recommended IPC placement over pleurodesis in patients with non-expandable lungs in the setting of a MPE [3]. However, the ATS made no further recommendations for the situation in which IPCs fail to appropriately drain effusions. As such, physicians have trialed various techniques, including the intrapleural administration of tissue plasminogen activator (t-PA) and dornase alfa (DNase) in this setting [4-6]. 

This case highlights the possible benefits of intrapleural administration of t-PA and DNase in a patient who presented with a complex right-sided MPE. Measures of improvement include symptomatic benefits and radiological findings in our patients.

## Case presentation

A 69-year-old Asian female with a past medical history of stage IV metastatic adenocarcinoma of the lung and a recurrent right-sided malignant pleural effusion presented with a chief complaint of dyspnea. Her dyspnea had progressively worsened over a three-day period since her last therapeutic thoracentesis was performed, which had removed 1.5 liters of serosanguinous fluid with mild symptomatic improvement. However, the patient noted worsening shortness of breath in the following days, prompting her return to the emergency department. She denied fevers or chills. Her only other complaint was a mild, non-productive cough. 

Five months prior to admission, our patient’s stage IV metastatic adenocarcinoma of the lung was diagnosed through a thoracentesis of a new right-sided pleural effusion, which was found to be positive for malignant cells on pleural fluid cytology. She additionally had two further thoracenteses performed over the three months following her initial diagnosis. These thoracenteses yielded exudative fluid. However, there was no growth observed on gram stains of these pleural fluid samples. Once the diagnosis of stage IV cancer was made, the patient established care with an oncologist who initiated chemotherapy with one cycle of carboplatin and one cycle of pemetrexed. Following this, her oncologist planned further treatment with seven cycles of ipilimumab and nivolumab to be administered over ten months. She had no significant surgical, family, or social history.

Her initial vital signs included a temperature of 98.2 °F, heart rate of 120 beats per minute, respiratory rate of 28 breaths per minute, blood pressure of 133/72 mmHg, and an oxygen saturation of 95% on two liters of oxygen delivered via nasal cannula. On the physical examination, the patient was found to be cachectic and in respiratory distress, with increased work of breathing. On lung auscultation, our patient’s left lung was clear; however, right-sided lung sounds were absent. Her heart rhythm was regular but tachycardic. Her abdomen was soft and nondistended. Her neurological examination was within normal limits.

**Figure 1:**
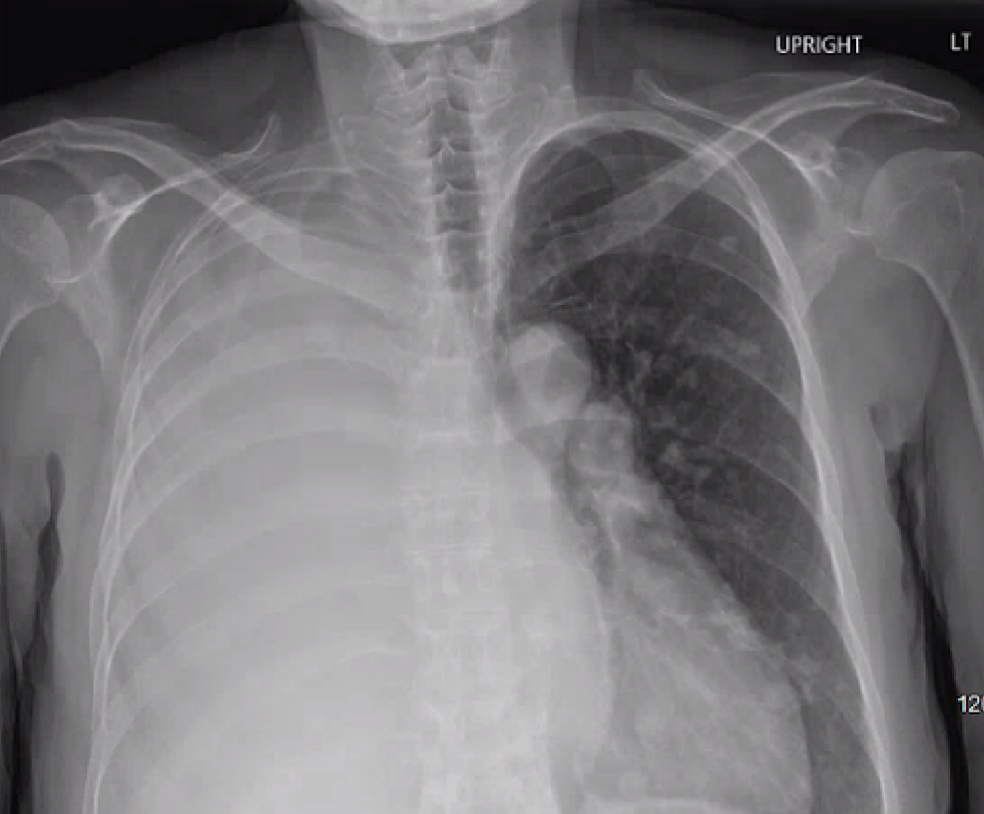
Chest radiograph during initial presentation demonstrating complete opacification of the right hemithorax with mediastinal shift to the opposite side.

**Figure 2:**
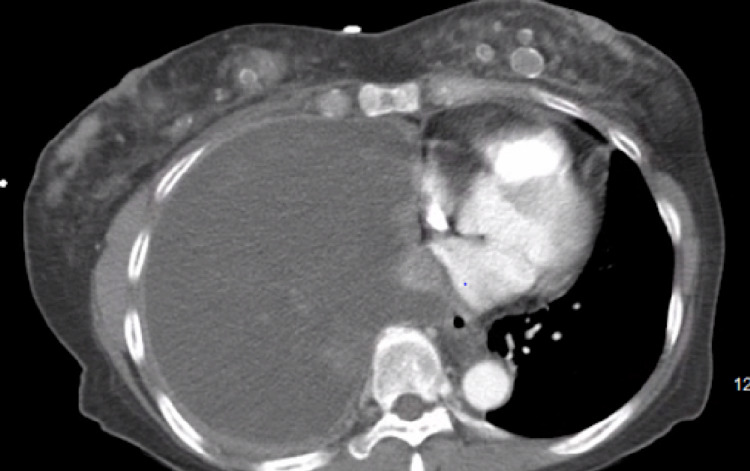
Computed tomography of the chest demonstrating a large right-sided pleural effusion with near-complete right lung collapse and a mediastinal shift of thoracic structures to the left.

In the emergency department, chest imaging was performed, which included a chest x-ray and a computed tomography (CT) scan of the chest. A chest x-ray showed complete opacification of the right hemithorax, suspicious for a large right-sided pleural effusion (Figure 1). These findings were confirmed by a CT scan of the chest, which revealed the aforementioned large right-sided pleural effusion, multiple pulmonary nodules, and mediastinal/hilar lymphadenopathy (Figure 2). Laboratory tests at the time of admission are shown in Table 1 and were only remarkable for leukocytosis and mild anemia.

**Table 1:** Initial laboratory findings significant for leukocytosis and anemia. g: gram, dL: deciliter, μL: microliter, mEq: milliequivalent, L: liter, mg: milligram, BUN: blood urea nitrogen.

	White blood cells (cells/μL)	Hemoglobin (g/dL)	Hematocrit (%)	Platelet (cells/μL)	Neutrophils (%)	Lymphocytes (%)
Reference values	4,300-11,100	11.5-15.5	36-46	120,000-360,000	38-75	20-48
Measured values	14,800	10.2	31.8	409,000	69	6
	Sodium (mEq/L)	Potassium (mEq/L)	Chloride (mEq/L)	BUN (mg/dL)	Creatinine (mg/dL)	Calcium (mg/dL)
Reference values	135-148	3.5-5.5	98-110	8-20	0.5-1.5	8.4-10.2
Measured values	134	4.2	97	12	0.8	9.5
	SARS-CoV-2 antigen					
Reference values	Negative					
Measured values	Negative					

Bedside ultrasonography of the lungs revealed a heavily septated, right-sided pocket of fluid surrounding the atelectatic lung. Given the presence of a complex MPE resistant to therapeutic thoracentesis, our pulmonary team decided to intervene by placing an IPC. The risks of IPC placement, such as bleeding, nerve damage, pneumothorax, and pneumothorax ex vacuo, were discussed with the patient prior to the procedure, and the patient gave informed consent. Following IPC placement at the bedside, there was an immediate evacuation of 700 milliliters (ml) of serosanguinous fluid. A repeat chest x-ray revealed appropriate placement of the IPC without improvement to the right-sided pleural effusion. Our patient continued to deny any symptomatic relief following IPC placement and fluid evacuation.

**Figure 3:**
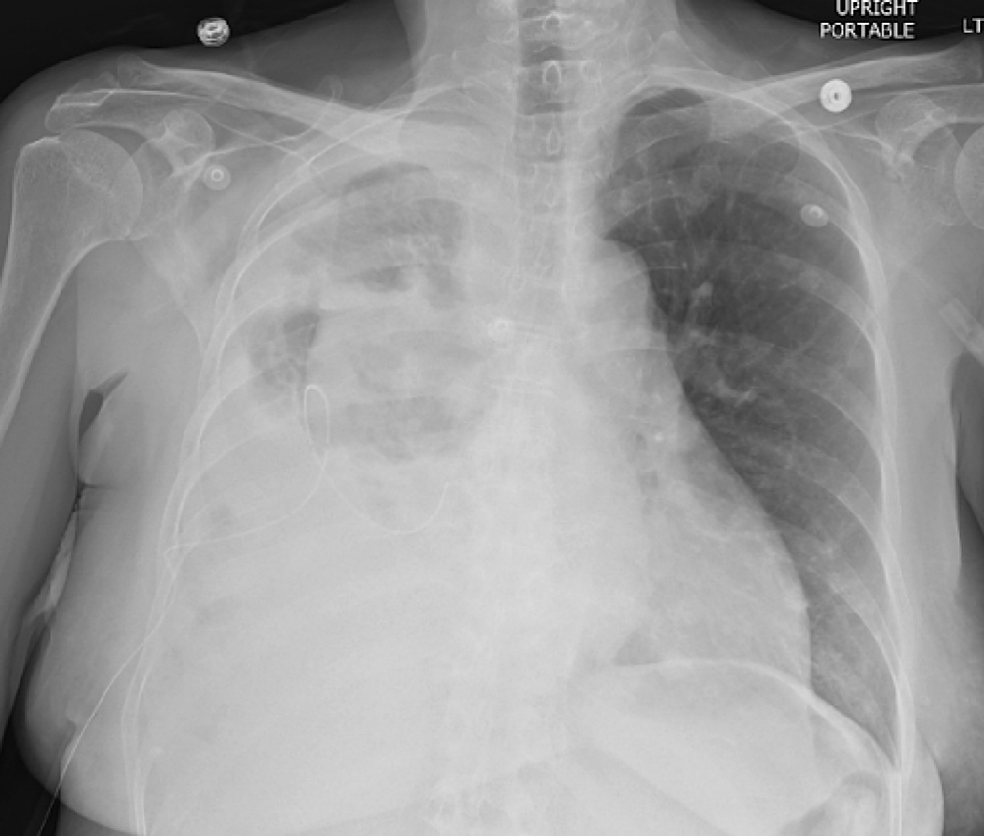
Chest radiograph 24 hours following initial t-PA and DNase administration through the IPC, demonstrating interval improvement in right-sided opacities. t-PA: tissue plasminogen activator; DNase: dornase alfa; IPC: indwelling pleural catheter.

The IPC was, thereafter, connected to suction for further drainage. Over the following 24 hours, less than 500 ml of fluid was drained from the catheter. Again, repeat imaging revealed no significant change, and the patient again denied significant symptomatic relief. Bedside ultrasonography re-demonstrated septations in the pleural effusion, which raised concern that the significant septations in the MPE were preventing appropriate drainage. Based on previous studies and failure of symptom improvement, the decision was therefore made to trial t-PA and DNase in an attempt to break down septations and more completely drain the patient’s MPE. We discussed the risks of bleeding and hemothorax with the administration of these medications, and the patient was agreeable to our plan despite these risks. The patient was scheduled for six doses of 10 milligrams (mg) of t-PA and 5 mg of DNase to be delivered by a pulmonologist through the IPC every 12 hours. The t-PA and DNase were drawn into two separate syringes and sequentially administered through the IPC, with flushes of 10 ml of normal saline in between each medication. Following the initial administration of these medications, there was an immediate increase in fluid output noted from the catheter. Twenty-four hours after the first administration of t-PA and DNase through the IPC, the patient drained a total of approximately 1200 ml of exudative fluid from the IPC, with improvement in her symptoms as well as improvement in radiographic findings, which are demonstrated in Figure 3.

**Figure 4:**
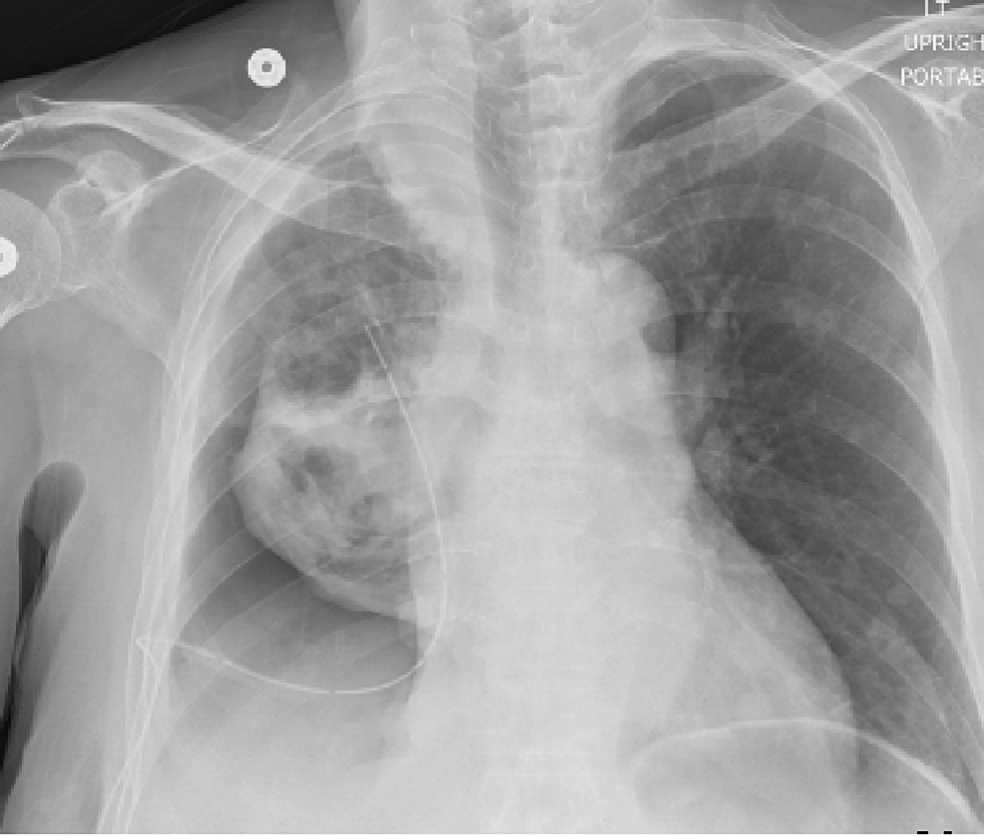
Chest radiograph following completion of t-PA and DNase therapy demonstrates marked improvement in right-sided opacities. t-PA: tissue plasminogen activator; DNase: dornase alfa.

Forty-eight hours following the initial administration of t-PA and DNase (a total of four doses thus far), an additional 665 ml of serosanguinous fluid was drained. At 72 hours after initiation of t-PA and DNase therapy (a total of six doses), an additional 750 ml of fluid was drained, yielding a total of 2,615 ml of fluid evacuated since the initiation of t-PA and DNase therapy. Once therapy was completed, a repeat chest x-ray was obtained with near-complete resolution of the patient’s right-sided MPE (Figure 4).

The patient was monitored for an additional 24 hours after completion of combination therapy to observe for any obvious potential adverse effects. After these 24 hours, the patient stated that her dyspnea had completely resolved and that she was having no residual effects related to IPC placement or therapy through the catheter. The patient was subsequently discharged home with the IPC in place and close follow-up through our pulmonary clinic.

## Discussion

Malignant pleural effusions are a common complication of various advanced neoplasms, most commonly lung cancer in men and breast cancer in women [1]. Ineffective and incomplete drainage of MPEs can be seen, particularly when the pleural space and pleural fluid become heavily septated or in the setting of highly viscous pleural fluid, as seen in MPEs. Seemingly, there is a positive correlation between the percentages of tumor cells and mitotic figures found in pleural fluid and the measured pleural fluid viscosity [7]. The mechanism by which MPEs septate may be explained by repeat pleural interventions. Repeated thoracentesis can lead to fibrin deposition, as invasive procedures may induce a local release of proinflammatory cytokines, vascular endothelial growth factor, and plasminogen activator inhibitor type 1 [8]. Our patient had three thoracenteses prior to her hospitalization, and these repeated interventions may have been a contributing factor to the significant septations and loculations noted, which may have led to ineffective drainage and, ultimately, a non-functional IPC.

Although the ATS 2018 guidelines recommend the placement of IPC over chemical pleurodesis for complex MPEs, no set recommendations have been made regarding the management of ineffective drainage through pleural catheters in this context. Video-assisted thoracoscopic surgery decortication may be useful in this setting, but as it is invasive, researchers have attempted to find less invasive alternatives. One possible solution theorized for ineffective drainage includes the use of intrapleural fibrinolytics. The 2nd Multicentre Intrapleural Sepsis Trial (MIST2 Trial) demonstrated the benefit of the use of intrapleural fibrinolytics (t-PA and DNase) in the context of infected complex pleural effusions [9]. However, this benefit was not extended to patients with complex MPEs. Since then, there have been a few studies evaluating the use of intrapleural fibrinolytics in the management of septated or complex MPE, with relatively variable results. The 3rd Therapeutic Intervention in Malignant Effusion Trial (TIME3 trial) studied the effects of 100,000 IU of pleural urokinase (three doses at 12-hour intervals) versus placebo in 71 non-draining MPE patients. This trial concluded that when fibrinolytics were administered through a pleural catheter, there were improvements in patients’ radiological outcomes, yet no statistically significant improvement in dyspnea was observed [10]. A second trial studied the use of intrapleural t-PA and DNase at one, two, or three doses in the context of 21 patients with complex MPEs. Results revealed improvements in the area of hemithoracic opacification on a chest x-ray, the volume of pleural fluid removed, and symptoms as delineated by the Borg rating of perceived exertion scale [6]. Based on the observations by Rivera-Flores et al. and the TIME3 trial, radiologic outcomes appeared to be improved with the intrapleural fibrinolytic agents; however, data regarding symptomatic improvement are inconsistent. This disparity in symptom improvement may be explained by the fact that River-Flores et al. employed the use of intrapleural DNase in conjunction with t-PA, which the TIME3 trial did not study. Currently, the data is limited and further studies are needed to determine the efficacy of intrapleural t-PA versus the combination of t-PA and DNase in the context of MPEs.

Our case report aims to contribute to the body of evidence favoring the use of t-PA and DNase in the management of complex MPEs. The patient in this report received six doses of t-PA and DNase with improvements noted in radiographic findings, symptomatic relief, as well as overall output from the pleural catheter. Although there is an inherent risk of bleeding and hemothorax with intrapleural fibrinolytic therapy, there were no obvious complications noted from the administration of these medications in our patient, which has been previously demonstrated by Rivera-Flores et al., MIST2, and TIME3. Our experience with intrapleural t-PA and DNase in the context of septated MPE was therefore safe and effective. Moving forward, it would be beneficial to see larger trials centering around the use of these medications and their dosing regimens for patients with complex MPEs.

## Conclusions

This case focuses on the utilization of t-PA and DNase in a patient with stage IV metastatic lung adenocarcinoma who presented with a loculated MPE. The current literature has demonstrated that intrapleural fibrinolytic administration is effective in reducing infectious complex pleural effusions. However, the study of its use in MPEs is limited to case reports and small studies. Currently, the evidence regarding the use of intrapleural fibrinolytics in the context of MPEs is inconsistent in regards to symptomatic improvement. Further, studies that specifically include the combination of t-PA and DNase are exceedingly rare. Our report demonstrates that intervention with intrapleural t-PA and DNase can safely provide significant radiologic and symptomatic improvement in complex MPEs. We hope that future studies can continue to investigate the potential beneficial use of intrapleural fibrinolytics in the context of complex MPEs.
